# Spontaneously immortalised bovine mammary epithelial cells exhibit a distinct gene expression pattern from the breast cancer cells

**DOI:** 10.1186/1471-2121-11-82

**Published:** 2010-10-22

**Authors:** Chenfu Zhao, Lu Meng, Hongyu Hu, Xudong Wang, Fangyu Shi, Yajuan Wang, Qianqian Li, Aixing Lin

**Affiliations:** 1 1 State Key Laboratory for Agrobiotechnology, College of Biological Sciences, China Agricultural University, Yuanmingyuan Xi lu, Haidian District, Beijing, 100193, China

## Abstract

**Background:**

Spontaneous immortalisation of cultured mammary epithelial cells (MECs) is an extremely rare event, and the molecular mechanism behind spontaneous immortalisation of MECs is unclear. Here, we report the establishment of a spontaneously immortalised bovine mammary epithelial cell line (BME65Cs) and the changes in gene expression associated with BME65Cs cells.

**Results:**

BME65Cs cells maintain the general characteristics of normal mammary epithelial cells in morphology, karyotype and immunohistochemistry, and are accompanied by the activation of endogenous *bTERT *(bovine Telomerase Reverse Transcriptase) and stabilisation of the telomere. Currently, BME65Cs cells have been passed for more than 220 generations, and these cells exhibit non-malignant transformation. The expression of multiple genes was investigated in BME65Cs cells, senescent BMECs (bovine MECs) cells, early passage BMECs cells and MCF-7 cells (a human breast cancer cell line). In comparison with early passage BMECs cells, the expression of senescence-relevant apoptosis-related gene were significantly changed in BME65Cs cells. P16^INK4a ^was downregulated, p53 was low expressed and Bax/Bcl-2 ratio was reversed. Moreover, a slight upregulation of the oncogene *c-Myc*, along with an undetectable level of breast tumor-related gene *Bag-1 *and *TRPS-1*, was observed in BME65Cs cells while these genes are all highly expressed in MCF-7. In addition, *DNMT1 *is upregulated in BME65Cs. These results suggest that the inhibition of both senescence and mitochondrial apoptosis signalling pathways contribute to the immortality of BME65Cs cells. The expression of *p53 *and *p16*^*INK4a *^in BME65Cs was altered in the pattern of down-regulation but not "loss", suggesting that this spontaneous immortalization is possibly initiated by other mechanism rather than gene mutation of *p53 *or *p16*^*INK4a*^.

**Conclusions:**

Spontaneously immortalised BME65Cs cells maintain many characteristics of normal BMEC cells and exhibit non-malignant transformation. Although this cell line displays altered patterns of gene expression, it is clearly distinct from malignant breast cancer cell line. It showed that co-inhibition of cellular senescence and mitochondrial apoptosis pathways coordinates BME65Cs cells immortalisation. Additionally, mechanisms other than gene mutation are likely to be involved in regulation of cellular functions. This study provides an insight into the relationship between cell senescence and immortalisation. BME65Cs cells will be useful in future studies of cellular senescence and tumorigenesis.

## Background

In serum-free culture, primary mammary epithelial cells (MECs) proliferate for 10 ± 20 population doublings (PD), after which they enter the first growth barrier: self-selection or M0 also named stasis [1]. Thereafter, small cells can appear, and these cells proliferate for up to 40-50 PD, after which they enter the second growth barrier: replicative senescence or M1, also called "agonescence" [2]. There are few reports of mammary epithelial cells (especially bovine MECs) spontaneously overcoming proliferative barriers and leading to immortalization [3].

Immortalisation is a process where cultured cells escape senescence and acquire the ability to grow in culture indefinitely [4]. Whether changes in gene expression accompany the spontaneous immortalisation of MECs is unclear, relevant data are available from cancer cells. It has been known that cellular replicative senescence is triggered by telomere shortening during each cell division [5]. Telomerase reverse transcriptase (TERT) maintains telomere length by replicating the telomere tracts and preventing cells from replicative senescence [6]. Overexpression of *TERT *has been used to immortalize a variety of normal diploid cell strains [7]. In addition, *TERT *gene has frequently been activated in cancer and stem cells [5,8]. The tumor suppressor p53, which is inactivated in numerous cancer cells due to its gene mutation [9,10], plays a key role in repressing transcription of *TERT *gene [11]. Another important tumor suppressor p16^INK4a^, a cyclin-dependent kinase (CDK) inhibitor, maintains normal cellular properties by preventing both centrosome dysfunction and genomic instability [12]. In mammary epithelial cells, it has been suggested that the pre-reduction of p16^INK4a ^by a direct or indirect method is required for *TERT*-mediated immortalisation [4]. In addition to the negative regulation of *p16*^*INK4a *^and *p53 *in the control of cell proliferation, some proto-oncogene such as *c-Myc *and others may help cells to escape senescence control and lead to carcinogenesis [11,13,14]. However, it is unclear whether the expression patterns of these cellular oncogenes and tumor suppressors are altered in immortalized cells and what is the mechanism for spontaneous immortalization of MECs.

Immortalised bovine mammary epithelial cell line can be used as an *in vitro *screening system to identify superior transgenes, and to improve genomic modification technological research, thereby improving the efficiency of transgenic animal production [15]. In this study, we described a spontaneously immortalised cell line (BME65Cs) derived from serial passages of bovine mammary epithelial cells. We performed a detailed investigation of cell characteristics and changes in gene expression in comparison to early passage cells, senescent cells and human mammary cancer cells (MCF-7). Our data showed that multiple signal pathways are involved in this process and BME65Cs is distinct from malignant MCF-7 in cancer associated gene expression.

## Results

### Spontaneously immortalised BME65Cs cells maintain the normal morphology and proliferation characteristic of normal BMECs

**Figure 1:**
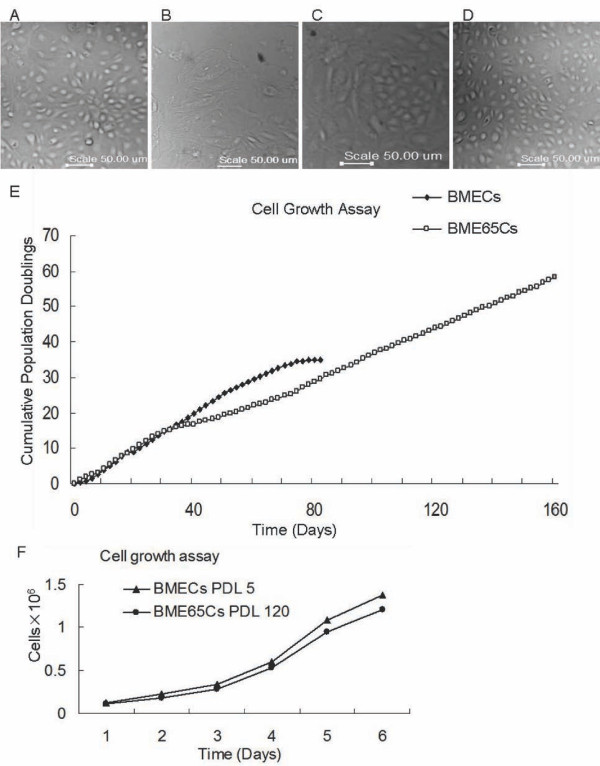
**Cell morphology and cell growth rate of BME65Cs cells**. **A**. Phase-contrast images of in vitro cultivated, early passage BMECs cells (PDL 10) with typical morphologies. **B**. Senescent BMECs cells (PDL 34) with an increased size. **C**. The initially isolated population of immortalised epithelial-like cells (BME65Cs). **D**. BME65Cs cells (PDL 120) with the typical cobblestone pattern of freshly isolated primary BMECs cells. Bars: 50 μm. **E**. Growth curve of BME65Cs and BMECs cells. To determine growth rates, primary (PDL 2) and immortal mammary epithelial cells (PDL 41) were plated at a density of 3 × 10 ^*5 *^cells/10 cm dish using culture conditions as described above. At 80% confluency, cells were trypsinised and subcultured every two days, and cumulative population doublings based on cell numbers was calculated. Normal BMECs cells entered the senescent state at PDL 34, and spontaneously immortalised BME65Cs colonies were generated at this stage. **F**. Growth rates of primary BMECs cells (PDL 5) and immortal BME65Cs cells (PDL 120). Cells were plated at a density of 1 × 10^*5 *^cells/10 cm dish for over 6 days of continuous culture, and cell numbers were counted each day. A similar increase in growth rate was observed between early passage MECs and immortalised BME65Cs cells.

The spontaneously immortalised BME65Cs cell line was established from *in vitro *cell culture. The initial cell focus was observed in a culture of BMECs cells when normal cells had entered the senescence state. This cell focus had a similar morphology to the early passage BMECs cells (Figure 1A) and was surrounded by an annulus of senescent cells (Figure 1C). In serum-containing culture, normal BMECs cells enter replicative senescence at PDL 34 (Figure 1B), whereas BME65Cs cells have been passed more than PDL 220. Continuous observation showed that before PDL 55 BME65Cs had a doubling time of approximately 48 hours, which was similar to normal cells. At PDL 55, although some of these cells become longer and less proliferative, a majority of cells remained epithelial morphology with a doubling time of approximately 72 hours (Figure 1D). This observation is consistent with the growth rate assay (Figure 1E and 1F). There was an adjustment phase required for BME65Cs cells to become a stable cell line. Interestingly, a small proportion of cells died in each passage.

### BME65Cs cells maintain many characteristics of normal mammary epithelial cells

**Figure 2:**
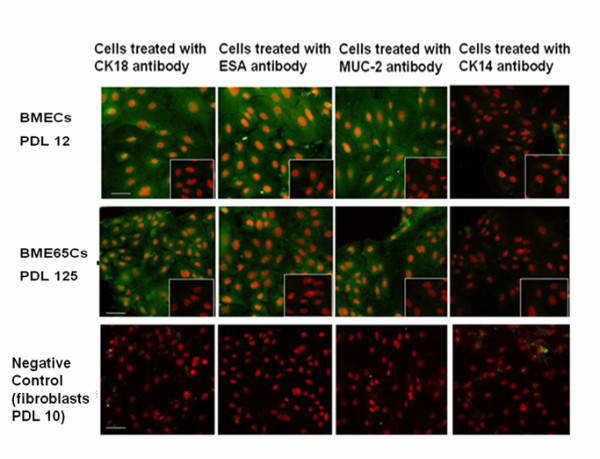
**Immunostaining of specific mammary epithelial cell protein markers**. Staining for CK18, ESA, MUC-2, and CK14 were observed in BMECs and BME65Cs cells, whereas staining for the isotype controls was faint (small pictures in bottom- right corner). PI was added as an immunofluorescence counterstain. Bars: 20 μm.

The breast epithelial compartment comprises two distinct lineages: the luminal epithelial and the myoepithelial lineage. It has been suggested that *in vitro*, normal luminal epithelial cells from the human mammary gland immunostain for CK18 (mammary epithelial cell specific marker protein cytokeratin 18), ESA (epithelial specific antigen) and MUC-2 (mucin 2 glycoprotein) [16], whereas myoepithelial cells are CK18^+^/ESA^-^/MUC^-^[16,17]. To test if BME65Cs cells are originated from the luminal epithelial or from myoepithelial, BME65Cs cells were immunostained with these three antibodies. The results showed that BME65Cs are CK18^+^/ESA^+^/MUC^+ ^in accordance with normal BMECs. Moreover, BME65Cs, similar to normal BMEC, showed a very slight signal in the staining of CK14, a maker of myoepithelial cells [1] (Figure 2).

**Figure 3:**
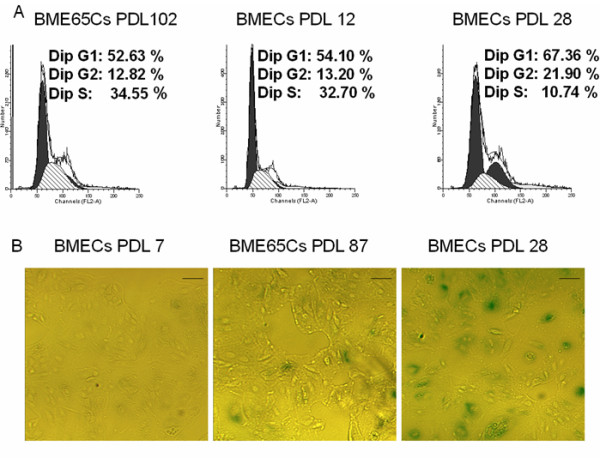
**Analysis of cell cycle and cell senescence-associated β-galactosidase activity in BME65Cs and BMECs cells**. **A**. Cell cycle analysis of BME65Cs cells (passage 102), BMECs cells primary passage 12 and 28 was performed using flow cytometry of propidium iodide-stained cells. A similar proportion of cells in S phase were observed in immortalised BME65Cs cells and early passage BMECs cells. **B**. Early passage 7, BMEC cells senescent passage 28 and immortal BME65Cs cells passage 87 were stained for β-galactosidase activity. Cells were fixed and incubated with X-gal. Blue staining indicates the presence of senescence associated β-galactosidase. This illustrates that a portion of immortalised BME65Cs cells (PDL 87) were in the senescent state. Bars: 20 μm.

Cell cycle and apoptosis are very important functional parameters used to assess cellular metabolism and physiology. We determined the cell cycle distribution of BME65Cs, early passage (PDL 12) and late passage (PDL 28) BMECs by flow cytometry. As shown in Figure 3A, growth arrest in the G0/G1 and G2/M phases was observed in late BMECs, but not in BME65Cs and early passage BMECs. Moreover, the proportion of cells in the S phase was increased in BME65Cs cells (PDL 102), but significantly decreased in late passage cells compared to early passage BMECs cells, suggesting that BME65Cs cell line has a higher proliferative capacity than that of normal cells.

To identify senescent state, a senescence marker, senescence-associated β-galactosidase (SA-β-gal), was examined in BME65Cs (PDL 87) as well as the control BMECs (PDL 28 and PDL 7). SA-β-gal staining was observed in late passage BMECs cells (PDL 28) and was not observed in early passage BMECs cells (PDL 7). As expected, approximately 10% of BME65Cs cells (PDL 87) stained for SA-β-gal (Figure 3B), consistent with the culture observation. This suggests that some of the immortalised BME65Cs cells still undergo senescence in normal culture conditions.

**Figure 4:**
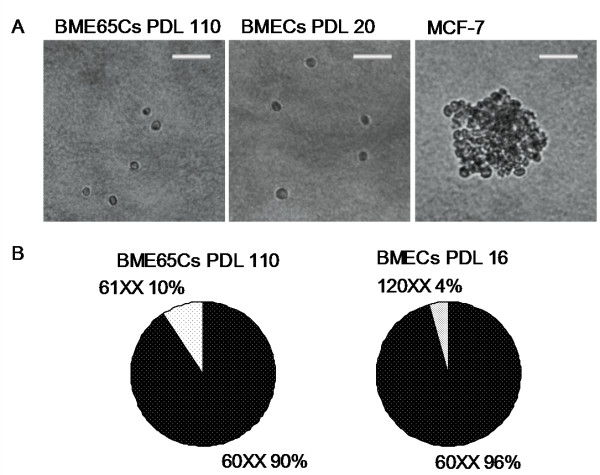
**Soft agar assay and karyotype analysis in immortal BME65s cells and early passage BMECs cells**. **A**. Analysis of colony formation used for evaluating malignant potential by the soft agar assay. Colonies containing more than ten viable cells were scored positive. Obvious colony formation was shown in MCF-7 cells, which served as a positive control. Bars: 50 μm. **B**. Comparison of chromosome numbers. At least 20 well-spread metaphase chromosomes from immortal BME65Cs cells (passage 110) and BMECs cells (primary passage16) were examined and the characteristic patterns illustrated were observed.

The growth of BME65Cs cells in serum-containing medium is dependent on the supplementation of insulin, epidermal growth factor and hydrocortisone, displaying normal BMECs cell growth characteristics. In comparison, the breast cancer cell line MCF-7 and the previously reported OMEC II (ovine immortal MECs) grow very rapidly in serum-containing medium without any further additives [18]. To determine whether immortalised BME65Cs cells have cancer cell potential, the soft agar assay for colony formation was performed. No colony was formed in BME65Cs (PDL 110) and normal BMECs (PDL 20), while obvious colony formation in MCF-7 is obvious (Figure 4A). Furthermore, the cell-cell contact in BME65Cs cells induced growth arrest (data not shown). These results together suggested that BME65Cs cells had a normal growth characteristic of anchor dependency and no tendency for malignant transformation [19].

To understand the cytogenetic property of the BME65Cs cell line, twenty-one metaphase spreads of PDL 110 were examined. Nineteen of them maintained normal female karyotype (60, XX), and only two cells appeared abnormal with 61, XX karyotype (Figure 4B). This differed from OMEC II, in which 3-4% of the cells were heteroploid at an earlier stage (PDL 5) and 25% at passage 60 [18]. Thus, the karyotype analysis indicated that the BME65Cs cell line has stable cytogenetic properties.

### Telomere length was stabilised without further elongation in BME65Cs cells

**Figure 5:**
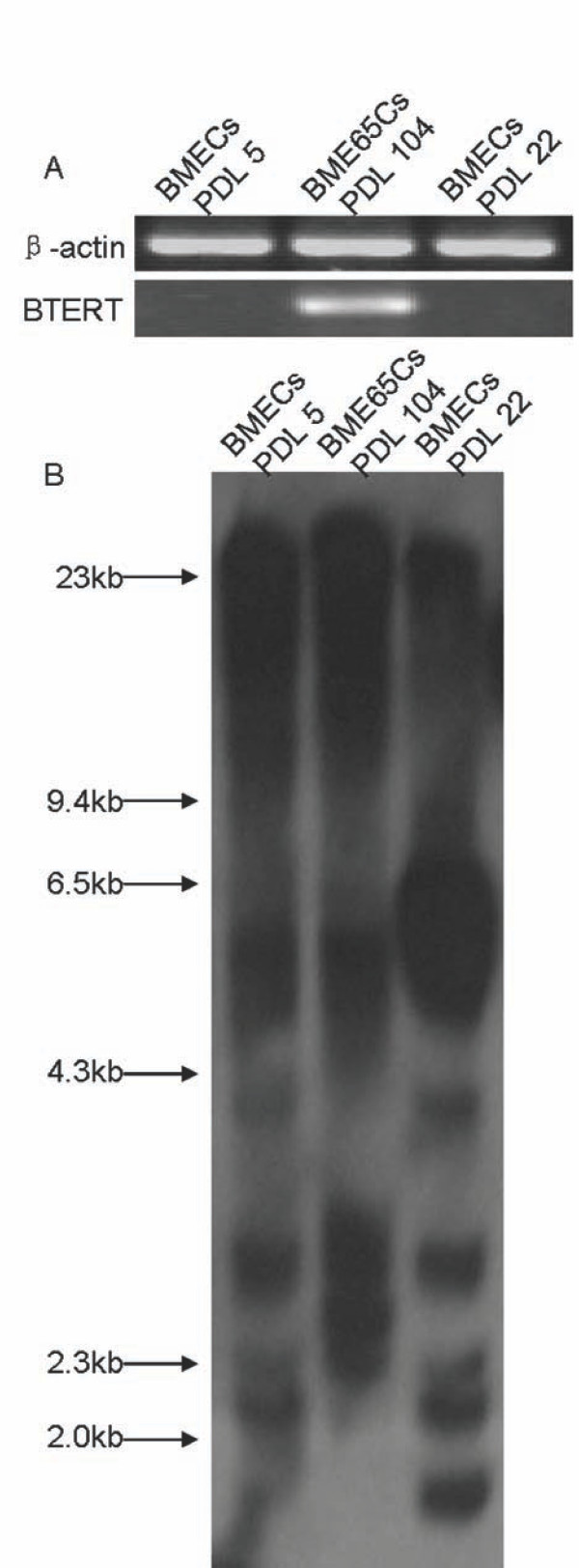
**Detection of *bTERT *expression and analysis of telomere length in BME65Cs and BMECs cells**. **A**. The detection of *bTERT *mRNA by RT-PCR. The bovine catalytic subunit of telomerase (*bTERT*) mRNA levels from primary early passage 5, BMECs cells senescent passage 22 and immortal BME65Cs cells passage 104 were determined by RT-PCR using sequence-specific primer pairs. *β-actin *was used as a loading control. **B**. Southern-blot analysis of mean telomeric length. Genomic Southern-blot analysis was performed to determine the telomeric lengths of primary early passage 5 and senescent passage 22 BMECs as well as immortal BME65Cs cells passage 104. The DNA marker (λ DNA/Hind III) was used to determine the telomere length of various samples.

*TERT *expression is thought to be an important factor for cellular immortalization [20]. To confirm whether endogenous *bTERT *is activated during immortalisation of BME65Cs cells, the expression of *bTERT *mRNA was detected by RT-PCR. As expected, *bTERT *was obviously transcribed in BME65Cs cells (PDL 104) but silenced in control BMECs cells (PDL 5 and 22) (Figure 5A).

Next, telomere lengths were further assayed by southern hybridisation. The mean telomere length of terminal restriction fragments (TRFs) in BME65Cs cells (PDL 104) is 18.7 kb. Comparatively, TRFs in BMECs cells at PDL 5 and PDL 22 are 17.9 and 6.4 kb, respectively (Figure 5B). This result suggests that telomere length is maintained in the BME65Cs cell line.

### Gene expression profile was changed in BME65Cs cells

**Figure 6:**
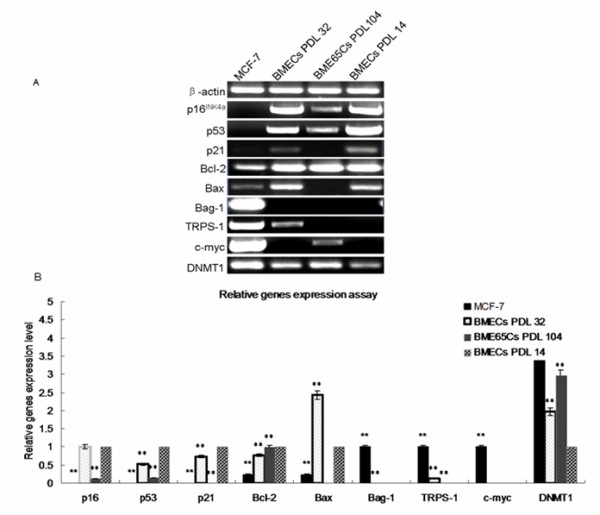
**Relative genes expression of early passage senescent BMECs, BME65Cs and MCF-7 cells**. **A**. The detection of relative genes expression by RT-PCR. **B**. Analysis of expression level by gray scale. The expression level of each gene in BMECs cells (PDL 14) was set to 1.0. The expression level for *TRPS-1*, *Bag-1 *and *c-Myc *in MCF-7 cells was set to 1.0 because these three genes expressions were not detected in BMECs cells (PDL 14)]. Other samples were adjusted accordingly. All PCR products were confirmed by sequencing. Gray scale analysis is from at least 3 independent experiments. *: P < 0.05 compared to BMECs cells passage 14 (Student's t-test). **: P < 0.01 compared to BMECs cells passage 14 (Student's t-test).

Cells that undergo immortalisation escape the controls of senescence and apoptosis. In order to gain insight into possible pathways involved in the BME65Cs cell line immortalisation, we selected 34 genes regulated mainly at the level of transcription to investigate the differences in gene expression between BME65Cs cells, senescent BMECs cells and early passage BMECs cells, as well as the MCF-7 cell line. Analysis of transcription profiles indicated that transcription levels of several genes, which are important for cell survival and growth, are significantly altered in BME65Cs comparing to the early passage BMECs (Figure 6).

It is well known that p16^INK4a ^and p53 are important for cellular apoptosis and senescence. Thus, we initially examined changes in expression of these two genes. Both p16^INK4a ^and p53 are significantly changed (8 fold decrease) in BME65Cs cells, indicating the importance of p16^INK4a ^and p53 signalling pathways in immortalisation of the BME65Cs cell line. Moreover, in senescent BMECs cells the expression of *p16*^*INK4a *^was unchanged, but there was a slight change in *p53 *(2 fold decrease), suggesting there is no correlation between the expression of *p53 *and *p16*^*INK4a*^. In MCF-7 cells, expression of both genes is undetectable (Row2-3 in Figure 6). As the p53 protein is frequently absent in cancer cells [9,10], we investigated p21^cip1^, one of the downstream targets of p53 that functions in cell cycle regulation [21], to enhance the result of p53. Paralleling the expression of *p53*, an undetectable signal of p21^cip1^was observed in both BME65Cs cells and MCF-7 cells, whereas a slight change (0.75 fold decrease) was observed in senescent cells (Row 4).

Next, we examined Bcl-2 family genes. Bcl-2 and Bax act crucial roles in the mitochondrial apoptosis pathway by suppressing and improving apoptotic cell death, respectively [22-25]. Expression of *Bcl-2 *mRNA was unchanged in BME65Cs cells and was slightly downregulated (0.75-fold) in senescent BMECs cells compared to early passaged BMECs cells, whereas expression of *Bcl-2 *was significantly lower (0.25 fold) in MCF-7 cells (Row 5). *Bax *mRNA was upregulated (2.5 fold) in senescent BMECs cells and downregulated (4 fold) in MCF-7 cells. Notably, *Bax *mRNA was not detected in BME65Cs cells (Row 6). Moreover, expression of another anti-apoptotic Bcl-2 family gene, Bag-1 [26], was unchanged in the three non-cancer cells. As shown, Bag-1 was undetectable in early passaged cells, senescent BMECs cells and BME65Cs cells, whereas there was high expression in MCF-7 cells (Row 7). Thus, the expression of *Bcl-2*, *Bax *and *Bag-1 *in BME65Cs cells was different from expression in MCF-7 cells. These results suggest that the immortalisation of the BME65Cs line involved inhibition of the mitochondrial apoptosis pathway.

To further understand the difference between spontaneously immortalised MECs and breast cancer cells, we investigated the expression of other genes considered to be differentially expressed or highly expressed in breast cancer cells. Two genes, *TRPS-1 *and *c-Myc *[27,14], displayed the difference. *TRPS-1 *expression was undetectable in both BME65Cs cells and early passage BMECs cells, which significantly distinguished from the high expression in MCF-7 cells. However, a low expression of *TRPS-1 *was shown in senescent cells (Row 8). Similarly, expression of *c-Myc *was almost undetectable in early passage and senescent BMECs cells. Although a low level of expression was detected in BME65Cs cells, by contrast, *c-Myc *was highly expressed in MCF-7 cells, about 200 fold higher than in BME65Cs cells (Row 9). Therefore, in addition to *Bag-1*, the expression level of *c-Myc *and *TRPS-1 *in BME65Cs cells was also markedly different from that in MCF-7 cells.

To understand the possible changes of DNA methylation that may cause altered gene expression in BME65Cs cells, we examined three DNA methyltransferases genes: *DNMT1, DNMT2 and DNMT3*. A significant difference was found for *DNMT1*. As shown, DNMT1 mRNA was upregulated in BME65Cs cells (3 fold), MCF-7 cells (4 fold) and senescent cells (2 fold). DNMT1 (DNA methyltransferase 1) is primarily a maintenance methylase, which reproduces DNA methylation patterns from hemimethylated DNA during cell division [28]. DNMT1 protein is disproportionately overexpressed in the S-phase fraction of estrogen receptor-negative cancer cell lines, including the MCF-7 line [29]. Thus, the upregulation of DNMT1 in the BME65Cs cell line suggests a possible change in DNA methylation.

## Discussion

We have established a spontaneously immortalised mammary epithelial cell line, BME65Cs. This cell line exhibits the majority of normal MECs features, whereas growth character, the ability to form colonies and expression of relevant breast tumor genes are significantly different from breast cancer cells (MCF-7). These data suggest that BME65Cs cells are not derived from malignant transformations. Whether or not *in vitro *spontaneous transformation is correlated with *in vivo *benign tumor transformation, the immortal BME65Cs cell line will be a useful tool for studying the molecular mechanism of tumorigenesis and cellular senescence. In contrast, *TERT *or SV40 gene mediated immortalization by the random integration of exogenous genes may bring an unforeseeable influence on natural gene expression and regulation.

There are three types of human mammary epithelial cell progenitors have been identified. The first is thought to be a luminal-restricted progenitor; the second type is a bipotent progenitor which is identified by its ability to produce "mixed"colonies in single cell assays; the third type of progenitor is thought to be myoepithelial restricted progenitor because it only produces cells with myoepithelial features [30]. In this study, a part of normal cultured BMECs show a basal (myoepithelial) markers (CK14^+^) coinciding with the observation in human MECs [1]. However, BME65Cs show a MUC^+^/ESA^+ ^CK14^- ^and no "mixed" population are found, which suggests BME65Cs is not derived from myoepithelial cell or bipotent progenitor. Although BME65Cs maintains many characters of luminal cells with stable proliferation behaviour over the long period of culture, it is unclear whether BME65Cs derived from a luminal epithelial lineage or from a progenitor cell with the capacity for luminal.

Transcriptional analysis of multiple genes sheds light on the number of genes involved in the immortalisation of BME65Cs cells. An interesting question raised by the data is the following: what are the main factors that contribute to initiation of the BME65Cs cells immortalisation? There are two signaling pathways, p16^INK4a^-pRb and p53-p21, that are mainly responsible for the control of cellular senescence [12,31]. The tumor suppressor genes *p16*^*INK4a *^and *p53 *are frequently inactivated in cancer cells; inhibition of them escapes cellular senescence leading to tumorgenesis [31,32]. In this study, a significant change (but not loss) of p16^INK4a ^and p53 were found in BME65Cs cells, suggesting that the inhibition of senescent-relevant pathways contributes to the BME65Cs cell line immortalisation. Additionally, the p16^INK4a ^-pRb pathway is thought to control the first growth barrier (self-selection) of MECs [1], and downregulation of *p16*^*INK4a *^is required for the *TERT*-mediated immortalisation of MECs [4]. Therefore, downregulation of *p16*^*INK4a *^is likely to be one of the key factors associating to BME65Cs cells immortalisation. However, there is no evidence suggesting that inactivation or downregulation of *p16*^*INK4a *^alone can induce immortalisation of MECs. Thus, changes in expression of other genes may also be involved in the initiation of BME65Cs cells immortalisation. Although the absence of p53 is thought to be an important initial factor for numbers of cancers, and several genes in the Bcl-2 family are regulated by p53 [9,24,31], it is unclear what the cause for the shortage of *p53 *expression (compare to normal cell) in BME65Cs cells. It has been known that telomere shortening and DNA damage activate p53 expression [21,24]. Thus, the maintenance of telomere length in BME65Cs cells may be the one reason for low expression level of p53. There remains a discrepancy between the relationship of p53 and p16^INK4a^. In most cancer cells, p16^INK4a ^and p53 are two-independent signalling pathways [33]. However, one report suggests that p16^INK4a ^regulates *p53 *in human mammary epithelial cells [34]. Despite this, the low expression of *p53 *might contribute to the coordination of BME65Cs cells immortalisation, because p53 plays a role in transcriptional repression of *TERT *[11]. Together, we speculate that the low expression of both *p16*^*INK4a *^and *p53 *is important for the maintenance of BME65Cs cells immortalisation.

Overexpression of *TERT *protects telomere repeats from erosion and, consequently, from induction of replicative senescence or apoptosis [35]. Thus, expression of *bTERT *may be another important factor for immortalisation of BME65Cs cells. *TERT *is positively regulated by c-Myc [36], but negatively regulated by p53 [11]. Therefore, the low expression of p53 and upregulation of c-Myc in BME65Cs cells might contribute to the activation of *bTERT*, and expression of *bTERT *may subsequently lead to stable telomere length in BME65Cs cells, aiding cells in overcoming senescence and leading to immortalisation. As a transcriptional factor, c-Myc is believed to regulate the expression of 15% of all genes, including genes involved in cell division, cell growth, and apoptosis [37]. In addition to the high expression in a number of cancer cells, *c-Myc *is also expressed in stem cells, and is thought to be a requirement for the maintenance of stemness [38]. Hence, we presume that the function pattern of cell linage-specific regulation of c-Myc might be dependent on its expression level. Curiously, the detection of TRF length shows discontinuity in BME65Cs. We suppose that it might be due to some non-immortal cells existing in BME65Cs, since some BME65Cs still died in each passage and showed SA-β-gal positive. It is interest to point out that the telomere length in BME65Cs is similar to that in early passage BMECs, this suggests immortalisation event for BME65Cs might occur in early passage cell.

In addition to *c-Myc*, two other genes, *TRPS-1 *and *Bag-1*, are also highly expressed in MCF-7 cells but undetectable in BME65C cells. *TRPS-1 *is called tricho-rhino-phalangeal syndromes type 1 gene, which mutations are previously shown to be associated with three rare autosomal dominant genetic disorders known as the tricho-rhino-phalangeal syndromes (TRPS). Recently, TRPS-1 protein has been found to be dramatically overexpressed in >90% of early and late-stage breast cancers [27]. Bag-1 is a multifunctional protein that interacts with a wide range of cellular targets and regulates cell survival, signaling, metastasis, proliferation and transcription [26,39]. In breast cancer, overexpression of *Bag-1 *has been detected in a majority of cases [40]. Taken together, the expression of *TRPS-1 *and *Bag*-*1 *may be used to evaluate malignant or non-malignant transformation of immortalised MECs. However, low expression of *TRPS-1 *was detected in senescent BMECs cells, implying that expression of *TRPS-1 *could be regulated by the cellular growth state, and TRPS-1 protein probably has other function that differs from its function in cancer cells.

Bcl-2 and Bax are important oncoproteins involved in regulation of cellular apoptosis via the mitochondrial apoptosis pathway. Bcl-2 functions in suppressing cell apoptosis, whereas Bax promotes cell apoptosis [23,41]. Paradoxically, expression of *Bcl-2 *has been consistently associated with a better prognosis of breast cancer patients [41-43]. It has been suggested that the Bax/Bcl-2 ratio determines whether apoptosis will occur [24]. In this study, Bax is deficient in BME65Cs cells, whereas Bcl-2 is highly expressed. Hence, immortalisation of BME65Cs cells is also accompanied by the inhibition of the mitochondrial apoptosis pathway. Interestingly, expression of another member of the Bcl-2 family, *Bag-1*, is undetectable in three different bovine MECs including BME65Cs cells and excluding MCF-7 cells.

Although we have analysed changes in expression of several genes from different cell strains, it is unclear what the initial mechanism is responsible for the change of these genes. Based on the fact that both *p16*^*INK4a *^and *p53-p21 *are low expressed but not silenced in BME65Cs cells, we propose that these two genes are not mutated during immortalisation. In addition, epigenetic control is another important mechanism in regulation of genes expression. Abnormal DNA methylation patterns have been shown in a number of cancer cells [44]. Overexpression of *DNMT1 *has been detected in several human cancers [45-47]. Carcinogenesis is often accompanied by increases of DNMT1 mRNA, DNMT1 protein, and DNA methyltransferase activity [48-50]. Moreover, epigenetic change has been shown the important role in immortalisation of human MECs [51,52]. In this study, DNMT1 is significantly increased in BME65Cs cells and highly expressed in MCF-7 cells, suggesting that DNA methylation status is likely to be linked to immortalisation. Whether the epigenetic changes conclusively lead to the altered expression of "immortalisation-initial genes" in BME65Cs cells requires further investigation.

## Conclusions

During the spontaneous immortalisation of mammary epithelial cells, BME65Cs cells undergo changes in gene expression. These changes improve cellular proliferation and may not contribute to malignant potential. The *bTERT *activation accompanied by the alternation of gene expression in multi-pathways contributes to BME65Cs immortalisation. In addition, changes in expression of immortalisation-related genes are likely to be controlled by some mechanisms other than gene mutation, such as DNA methylation. This study provides an insight into the relationship between cellular senescence and immortalisation. However, more research is required to provide a more detailed mechanism for the initiation of BME65Cs cells immortalisation. Regardless, this spontaneous immortal cell line will be a useful tool for studying cell molecular biology, in addition to the investigation of mammary gland specific expression and somatic gene targeting technology.

## Methods

### Cell culture

Mammary gland tissues were obtained from a normal two-year old Chinese Holstein dairy cattle in lactation period by surgical operation. Several small pieces of tissue were carefully dissected from the inner part of the large mammary gland tissue, and enzymatically digested by a combination of trypsin, hyaluronidase (sigma) and collagenase (sigma) to separate breast epithelial and ductal tissue from stromal cellular components. Cells were planted in serum-containing medium: DMEM-F12 supplemented with 10% iron-supplemented fetal cattle serum, 5 μg of insulin per ml, 10 ng of epidermal growth factor per ml, 0.5 μg of hydrocortisone per ml, and 100 units/ml penicillin and streptomycin. Digested cells were cultured at 37°C in a CO_2 _incubator. The primary BMECs cells were passaged at a 1: 8 split, and thus each passage represented three population doublings. Population doublings (PD) were calculated by the following equation: PD = [ln (split ratio)/ln2]. The population doubling level (PDL) resulted as the cumulative PD. BMECs cells displaying typical cobblestone morphology were subcultured at or near confluence, and the cumulative PDL was recorded. Immortalised BME65Cs cells were derived from cultured cell over PDL 34 and continuously cultured in the same medium. MCF-7 cells were cultured in DMEM medium supplemented with 10% iron-supplemented fetal cattle serum.

### Cell growth assay

To determine growth rates, primary and immortal mammary epithelial cells (PDL 41) were plated at a density of 3 × 10^5 ^cells/10 cm dish using culture conditions as described above. At 80% confluency, cells were trypsinised and subcultured every two days, and cumulative population doublings based on cell numbers was calculated. Cell growth was also determined by plating cells at a density of 1 × 10^5 ^cells/10 cm dish using conditions described above, and cells were counted each day for up to 6 days to determine cell numbers.

### Indirect immunofluorescent staining

BMECs (PDL 12), BME65Cs (PDL 125) and fibroblast cells (PDL 10) were seeded in gelatin-coated chamber slides. Cells were fixed and permeabilised by addition of 3% paraformaldehyde for 30 minutes, followed by incubation with 0.3% Triton X-100 in PBS for 20 min. After blocking with 10% FCS in PBS, cells were incubated with the mouse anti-human cytokeratin 14 (CK14) antibody, the mouse anti-human cytokeratin 18 (CK18) antibody, the epithelial specific antigen (ESA) antibody and the mucin 2 glycoprotein monoclonaly (MUC-2) antibody, respectively, followed by incubation with the secondary antibody (goat anti-mouse IgG-fluorescein isothiocyanate (FITC)-conjugated antibody, Sigma). Thereafter, 1.5 mM PI was added as a counterstain for immunofluorescence and incubated with cells in the dark for 5 minutes at room temperature. The mouse IgG3, IgG1 and IgG2a were used as isotype controls, respectively. Specific fluorescence was detected by using a laser confocal microscope.

### Flow cytometric analysis

BMECs cells at PDL 12 and PDL 28, and BME65Cs cells (PDL 102) were plated at 10^5 ^cells/well on 35mm plates in corresponding medium. Cultured cells were washed with PBS solution and trypsinised, then fixed in an ethanol (80%)/PBS solution at 4°C for 30 min. Thereafter, cells were incubated in a solution containing 0.2 mg/ml RNase at 37°C for 30 minutes and stained with 50 ng/ml propidium iodide at room temperature for 30 minutes, and analysed by flow cytometry (FACScaliber; Becton Dickinson, San Jose, CA).

### Senescence-associated β-galactosidase assay (SA-β-gal)

BMECs cells at PDL 7 and PDL 28, and BME65Cs cells (PDL 87) were washed with PBS and fixed for 5 minutes at room temperature with 3% formaldehyde. Subsequently, staining was performed with X-gal solution (1 mg/mL X-gal, 0.12 mM K_4_Fe [CN]_6_, 0.12 mM K3Fe [CN]_6_, 1 mM MgCl_2 _in PBS at pH 6.0) for 12h at 37°C and then washed with PBS and photographed.

### Soft agar assay

Soft agar assays were performed in duplicates using 300 cells/cm^2 ^as previously described [19]. MCF-7 cells were used as a positive control. The ability for anchorage-independent growth shown by clone formation was analysed after three weeks. Clones containing more than ten viable cells were scored as positive.

### Chromosomal analysis

Metaphase chromosomes were prepared from BMECs cells (PDL 16) and BME65Cs cells (PDL 110), and grown on plastic plates. Logarithmically growing cells were arrested in metaphase using colcemid (sigma) (100 ng/ml), trypsinised, incubated for 30 minutes in 75 mM KCl, fixed with methanol:acetic acid (3:1), dropped onto cold glass slides and stained with Giemsa (sigma). At least 20 well spread metaphases chromosomes from cultures were examined to obtain the characteristic patterns illustrated.

### Terminal restriction fragment (TRF) analysis

The mean telomere length was evaluated by using TRF analysis, a variation of standard Southern analysis, and was quantified. Genomic DNA was extracted from BME65Cs cells (PDL 104), BMECs cells at PDL 5 and PDL 22. Two micrograms of DNA was digested with a Hinf I/Rsa I mixture (10 U each) and separated on a 0.6% agarose gel by electrophoresis. To ascertain that a comparable amount of DNA from individual cells was loaded in each lane, the gel was stained with ethidium bromide and examined by UV light. The gel was then depurinated for 15 min in 0.25 M HCl, denatured for 20-30 min in 0.4 M NaOH and transferred to a nylon membrane using 0.4 M NaOH as the transferring buffer. After rinsing in 2×SSC and neutralisation solution, hybridisation was performed overnight at 65°C using a specific biotinylated detection telomere probe, then washed twice (5 minutes each time) in 2×SSC/0.1% sodium dodecyl sulfate (SDS) at room temperature and one time for 15 minutes in 0.2×SSC/0.1% SDS. The Luminescence Detection Kit (Roche) was used to detect TRF DNA on Hyperfilm. The size (in kilobases) of the mean telomere length was determined by comparing the migration distance with fragments of known DNA markers.

### Real-time PCR measurements of relative mRNA levels

mRNA was collected from early passage (BMECs PDL 14), senescent (BMECs PDL 32) and spontaneously immortalised (BME65Cs PDL 104) mammary epithelial cells, as well as MCF-7 cells. Total cellular RNAs were extracted with an RNA extraction kit (Tiangen Co.) and 5 μg of total RNA from each sample was used for reverse transcription. One microliter of reverse transcription product was used as template for PCR amplification. Relative quantification was evaluated by calculating the ratios of copy numbers between target genes and the reference *β-actin *gene (a housekeeping gene). Primer pairs used for the analysis are displayed in Table 1. Considering the mRNA and protein levels in eukaryotic cells are poorly correlated [53], genes with a high correlation between mRNA and protein shown in previous reports were selected for detection (data not shown).

**Table 1:** Primer pairs used in the real-time PCR experiments of gene expression profiles Primer pairs applied for real-time PCR

Gene	Accession	Sense primer	Antisense primer	Product
bTERT	NM001046242	TCACGGGTCAAGACGCTGT	CGGGCATAACTGGAGTGGT	886 bp
p16^INK4a^	XM868375	GGTGATGATGATGGGCAGCG	ACCAGCGTGTCCTGGAAGC	134 bp
p53	NM174201	CGGAACACCTTTAGACACAGT	GTAGGCAGTGCTCGCTTAGT	304 bp
p21^Cip1^	NM001098958	TCTACCACTCCAAACGCA	GCAAAGGATGAAGAGGGTT	331 bp
TRPS-1	NM014112*	GCCTTCTTTGAGTTCGGA	CCCTCTGCTGTTTGTTGAG	384 bp
Bcl-2	XM586976	GCCTTCTTTGAGTTCGGA	TTCAGAGACAGCCAGGAGA	209 bp
Bax	NM173894	AGTGGCGGCTGAAATGTT	TTCTTCCAGATGGTGAGCG	287 bp
Bag-1	NM001076291	TGCCATTGTCAGCACTTG	TGTTCTGTTCCACTGTGTCAC	376 bp
c-Myc	AF519455	GTGTCTACCCATCAGCACAA	AACTGTTCTCGCCTCTTCTG	376 bp
DNMT1	NM182651	CCCGAGTGTGGAAAGTGTA	GAACATCTGCCCATTGCT	392 bp

*TRPS-1 primer pairs were designed by comparing homologous sequences between the *Homo sapiens *and *Bos taurus *genome, because the cattle *TRPS-1 *sequence has not been reported.

All other primer sequences were cited from GenBank. Each pairs of primers span intron-extron boundaries.

## Competing interests

The authors declare that they have no competing interests.

## Authors' contributions

CZ performed the isolation of cells, cell culture, cell growth analysis, cell cycle analysis, soft agar assay, chromosomal analysis and RT-PCR analysis, and participated in conceiving and designing the study and drafted the manuscript. LM performed the telomere length analysis and assisted in cell culture and RT-PCR analysis. HH and YW performed the senescence-associated β-galactosidase assay and assisted in chromosomal analysis. XW and QL performed the immuno-histochemical studies with FS. AL coordinated the study, and participated in conceiving and designing the study and helped draft the manuscript. All authors read and approved the final manuscript.
